# RETRACTED: Frolli et al. Universal Design for Learning for Children with ADHD. *Children* 2023, *10*, 1350

**DOI:** 10.3390/children13081037

**Published:** 2026-08-04

**Authors:** Alessandro Frolli, Francesco Cerciello, Clara Esposito, Maria Carla Ricci, Rossana Pia Laccone, Fabio Bisogni

**Affiliations:** 1 Disability Research Centre of Rome University of International Studies, 00147 Rome, Italy; francesco.cerciello@unint.eu (F.C.); clara.esposito@unint.eu (C.E.); rossana.laccone@gmail.com (R.P.L.); fabio.bisogni@unint.eu (F.B.); 2 FINDS—Italian Neuroscience and Developmental Disorders Foundation, 81040 Caserta, Italy; m.ricci@unint.eu

The journal retracts the article titled “Universal Design for Learning for Children with ADHD” [1], cited above.

Following publication, concerns were brought to the attention of the Editorial Office regarding the data sample and the ethical approval information reported in this publication [1].

In accordance with the journal’s standard procedures, the Editorial Office and Editorial Board conducted an investigation during which questions were raised about the data sample and the lack of appropriate Institutional Review Board approval prior to the participants’ enrolment. The authors cooperated with the investigation and provided additional information relating to the data sample and ethical approval. However, after careful consideration, the Editorial Board determined that the explanations and supporting documentation did not sufficiently address the remaining concerns about the ethical approval information and that the study is non-compliant with MDPI’s policies for Research Involving Human Subjects. As a result, the Editorial Board has decided to retract this paper as per MDPI’s retraction policy.

This retraction was approved by the Editor-in-Chief of the *Children* journal.

The authors have been informed of this retraction and indicated their disagreement with this decision.
